# Supplementary material for: Stability and molecular pathways to the formation of spin defects in silicon carbide
Source: Nat Commun. 2021 Nov 3;12:6325. doi: 10.1038/s41467-021-26419-0 (PMC8566517; doi:10.1038/s41467-021-26419-0)
Supplement: Supplementary file 1 — Supplementary Information [file 41467_2021_26419_MOESM1_ESM.pdf]

Supplementary Information for

Stability and molecular pathways to the formation of spin defects in silicon carbide

Elizabeth M.Y. Lee, Alvin Yu, Juan J. de Pablo*, and Giulia Galli*

*Corresponding authors: depablo@uchicago.edu, gagalli@uchicago.edu

Supplementary Note 1: Comparisons between density functional theory (DFT) and classical empirical force field results

A. Finite-size and thermal effects on free energies of defect migration in classical molecular dynamics (MD)

Based on enhanced sampling simulations with two different supercell sizes (see Supplementary Table 1), we find that the free energy of migration for a single vacancy can vary from ~0.1-0.2 eV, which is comparable to the thermal energy ($k_B T$~0.12 K at T =1500 K).

Estimated migration barriers at 0 K indicate that the thermal effect may be as large as ~0.3 eV, nevertheless smaller than the difference in free energy barrier between the V_{Si} and V_C migration processes (~1.0 eV) (see Supplementary Table 1).

Supplementary Table 1: Size and thermal effects in potential of mean force (PMF) calculations with classical MD

Process	Initial State	Final State	Classical MD free energy barrier at 1500 K for a 4096-atom supercell (eV)	Classical MD potential energy barrier at 0 K for a 4096-atom supercell* (eV)	Classical MD free energy barrier at 1500 K for a 216-atom supercell (eV)
V_C migration	V_C	V_C'	2.5	2.78	2.6
V_{Si} migration	V_{Si}	V_{Si}'	3.5	3.67	3.7

* Computed using the climbing-image nudge elastic band method as implemented in the LAMMPS software package.

B. Free energy landscapes of defect transformation mechanisms from first principles molecular dynamics (FPMD) and classical MD using enhanced sampling simulations

Supplementary Table 2: Comparisons between FPMD and classical MD results

Process	Initial state	Final state	Classical MD free energy barrier, forward (eV)	Classical MD free energy difference (final – initial state), (eV)	FPMD free barrier, forward (eV)	FPMD free energy difference (final – initial state), (eV)
V_C migration	V_C	V_C'	2.5	0	3.9	0
V_{Si} migration	V_{Si}	V_{Si}'	3.5	0	3.0	0
Vacancy-antisite conversion	V_{Si}	$C_{Si}V_C$	1.3	-0.6	1.3	-1.3
VV dissociation via V_C	VV	V_{Si} + V_C	2.2	0.9	3.8	1.5

migration						
VV dissociation via V_{Si} migration	VV	V_{Si} + V_C	-	-	3.0	1.5
VV reorientation via V_C migration	VV	VV'	1.7	0	2.5	0
VV reorientation via V_{Si} migration	VV	VV'	-	-	3.1	0

We compared the energetics of vacancy defects using two different approaches: empirical force fields and DFT. In order to do so, we performed enhanced sampling simulations with classical MD and first principles MD (FPMD) based on DFT (with the PBE functional) at 1500 K, and we computed the free energy surfaces for the following processes: monovacancy migration, VV formation, and VV reorientation. The barriers and energy differences between the initial and the final states are summarized in Supplementary Table 2.

We find quantitative differences between the results of classical MD and FPMD, but overall the results are in qualitative agreement. Specifically, VV has a lower free energy than the dissociated states (*i.e.*, those of the V_{Si} and V_C); $C_{Si}V_C$ has a lower free energy than V_{Si}; and the V_{Si}-to-$C_{Si}V_C$ conversion process has a lower free energy barrier than the single vacancy (V_{Si} or V_C) migration processes. Also, the free energy barrier for VV reorientation is lower than that of VV dissociation into V_{Si} and V_C. Therefore, the key findings of our work—(1) VV formation is limited by V_{Si} stability and (2) VV reorientation occurs without dissociation—are now also confirmed by using FPMD simulations.

A notable difference between classical and FPMD results is that the empirical force field predicts V_C migration barrier to be lower than that of V_{Si}, while the opposite is true using DFT.

Supplementary Note 2: Kinetic Monte Carlo simulations

A minimal model to simulate the population dynamics of vacancies based on the energetics from DFT was constructed using a kinetic Monte Carlo (KMC) algorithm. To simulate vacancy migration processes occurring at $T \geq 1500$ K (Figures 4b and 5a in the main text), we considered the following processes (P1 through P8):

P1. VV → V_{Si} + V_C via V_{Si} migration
P2. V_{Si} + V_C → VV via V_{Si} migration
P3. $C_{Si}V_C$ → V_{Si}
P4. V_{Si} → $C_{Si}V_C$,
P5. $C_{Si}V_C$ → C_{Si} + V_C
P6. C_{Si} + V_C → $C_{Si}V_C$
P7. VV → V_{Si} + V_C via V_C migration
P8. V_{Si} + V_C → VV via V_C migration

Processes P2, P4, P6, and P8 are the reverse of P1, P3, P5, and P7, respectively.

For each Monte Carlo trajectory, all possible transition pathways are computed from the starting state. The rate expression for each process, from states *i* to *j*, is given by $r_{ij} = k_{ij}C_i$ where C_i is the concentration of species *i*; $k_{ij} = k_{0,ij}\exp\left[-\Delta E_{ij}{}^{\neq}/k_B T\right]$ is the rate constant; $k_{0,ij}$ is the rate constant prefactor; $\Delta E_{ij}{}^{\neq}$ the energy barrier; k_B the Boltzmann factor; and T the temperature. We assumed that the prefactor is the same for all processes: $k_{0,ij} = k_0$. Once rates are tabulated, the *n*-th process is randomly selected by finding *n* for which $R_{n-1} < u_1 R_N \leq R_n$, where $R_n = \sum_{j=1}^{n} r_j$ is the cumulative function; $R_N = \sum_{j=1}^{8} r_j$ is the total reaction propensity; and $u_1 \in (0,1]$ is a random number drawn from a uniform distribution. Selected process from states *i* to *j* is carried out by decreasing the count of each species *i* by 1 and increasing the count of each species *j* by 1. Time *t* is updated by $t = t + \Delta t$ where $\Delta t = -\ln u_2/R_N$ and $u_2 \in (0,1]$ is another random number drawn from a uniform distribution. This process is repeated until $t = 10^{17} k_0{}^{-1}$, unless told otherwise. Each trajectory began with 2000 vacancies with a total vacancy concentration of 1e-3 % (or one vacancy for every 10,000 atoms). 50,000 independent trajectories were sampled.

In this simple KMC model, processes involving carbon atom migration (P3/P4, P5/P6, and P7/P8) have the same energy barrier as that of V_C migration (3.9 eV), while those involving silicon atom migration (P1/P2) have the same barrier as that of V_{Si} migration (3.0 eV), as computed from FPMD simulations (Supplementary Table 2). The relative energy differences between the initial and final states for P1/P2, P3/P4, and P7/P8 are also parameterized based on our FPMD results (Supplementary Table 2). For P5/P6, the energy difference between the $C_{Si}V_C$ and C_{Si} + V_C states is estimated to be ~0.2 eV from the deep level transient spectroscopy study[1] of $C_{Si}V_C$, which measures the decay of bound $C_{Si}V_C$ with increasing annealing temperature (see Supplementary Fig. 8). KMC input parameters are summarized in Supplementary Table 3.

Supplementary Table 3: KMC input parameters

Process	Rate expression, r_i	Energy barrier, $\Delta E^{\neq}$ (eV)
P1	$r_1 = k_1 C_{VV}$	3.0
P2	$r_2 = k_2 C_{V_{Si}} C_{V_C}$	3.0 – [E(V_{Si} + V_C) – E(VV)] = 1.5
P3	$r_3 = k_3 C_{C_{Si}V_C}$	3.9
P4	$r_4 = k_4 C_{V_{Si}}$	3.9 – [E(V_{Si}) – E($C_{Si}V_C$)] = 2.6
P5	$r_5 = k_5 C_{C_{Si}V_C}$	3.9
P6	$r_6 = k_6 C_{C_{Si}} C_{V_C}$	3.9 – [E(C_{Si} + V_C) – E($C_{Si}V_C$)] = 4.1
P7	$r_7 = k_7 C_{VV}$	3.9
P8	$r_8 = k_8 C_{V_{Si}} C_{V_C}$	3.9 – [E(V_{Si} + V_C) – E(VV)] = 2.4

Supplementary Note 3: Thermal quenching of new spin defects consisting of antistites and vacancies using classical MD simulations

At temperatures above ~1800 K, VV dissociated into [$C_{Si}V_C$ and V_C]$_n$ for n=1,2,… in classical MD simulations. To determine if these antisite-vacancy complexes could be stabilized by thermal quenching, we performed additional classical MD simulations starting from [$C_{Si}V_C$ and V_C]$_{n=1}$ and [$C_{Si}V_C$ and V_C]$_{n=3}$, in which temperature was continuously decreased from 1800 K to 300 K at the NVT ensemble with a Berendsen thermostat using a temperature damping parameter of 100 fs. For a range of cooling rates (0.1, 0.5, and 1 K/ps), [$C_{Si}V_C$ and V_C] species remained stationary during the cooling process, suggesting that these defects can be stabilized by thermal quenching.

Supplementary Note 4: Size-dependence of DFT calculations

To ascertain the finite size effect in migration energies obtained from DFT calculations, we evaluated the total energy difference between divacancy and monovacancies that are separated by a single Si-C bond (V_{Si} + V_C). For each defect state, the DFT total energy was minimized for a ground state triplet configuration that is neutrally charged. The result is summarized in Supplementary Table 4.

Supplementary Table 4: DFT-PBE size dependence

Supercell size	E(VV) – E(V_{Si} + V_C)
216-atom	1.98
512-atom	2.04

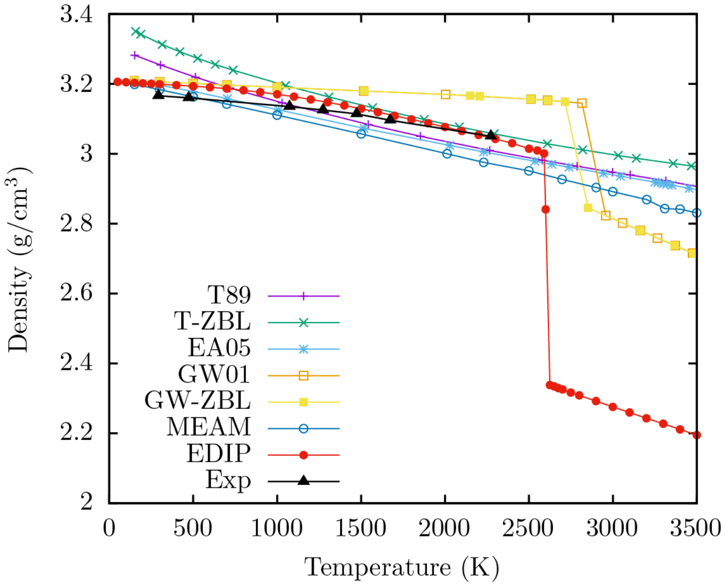

Supplementary Figure 1: Empirical interatomic potential benchmark study. Density of 3C-SiC at a given temperature was computed from a 1 ns-long classical MD simulation performed in the NPT ensemble at 1 atm using 4096 atom cells. The black line corresponds to the experimental data from Ref. 2. The following empirical force fields were used: "T89" (in purple) for the Tersoff potential[3,4], "T-ZBL" (in green) for the Tersoff potential with the Ziegler-Biersack-Littmark (ZBL) screened nuclear repulsion[5], "EA05" (in light blue) for the Erhart-Albe potential[6], "GA01" (in orange) for the Gao-Weber potential[7], "GW-ZBL" (in yellow) for the Gao-Weber potential with the ZBL screened nuclear repulsion[8], "MEAM" (in dark blue) for the modified embedded-atom method[9], and "EDIP" (in red) for the environment-dependent interatomic potential[10]. Among these force fields, EDIP was chosen for this study based on its reasonable agreement with experimental data on the temperature-dependent density up to ~2200 K and the decomposition temperature, e.g., T_m~2620 K using EDIP and 2818 K from experiments[11].

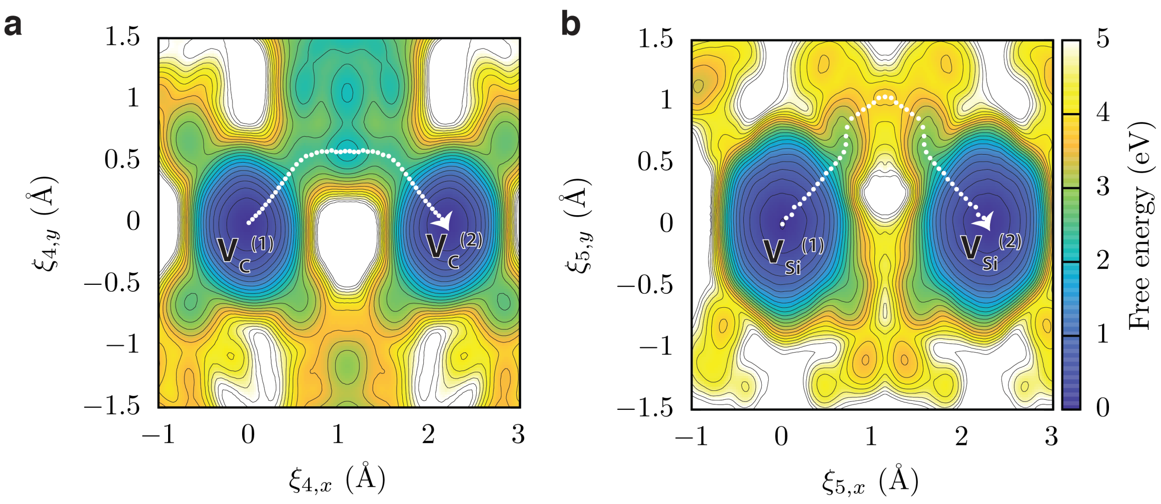

Supplementary Figure 2: Free energy surface for monovacancy migration from classical MD. a, Two dimensional (2D) potential of mean force (PMF) of V_C migration via diffusion of a nearest carbon atom, whose cartesian coordinates are used as the collective variables. **b**, 2D PMF of V_{Si} migration via diffusion of a nearest silicon atom, whose cartesian coordinates are used as the collective variables. The mean free energy paths (MFEPs) are shown as white dotted lines. Free energy barriers for migration are ~2.5 eV and ~3.5 eV for V_C and V_{Si}, respectively.

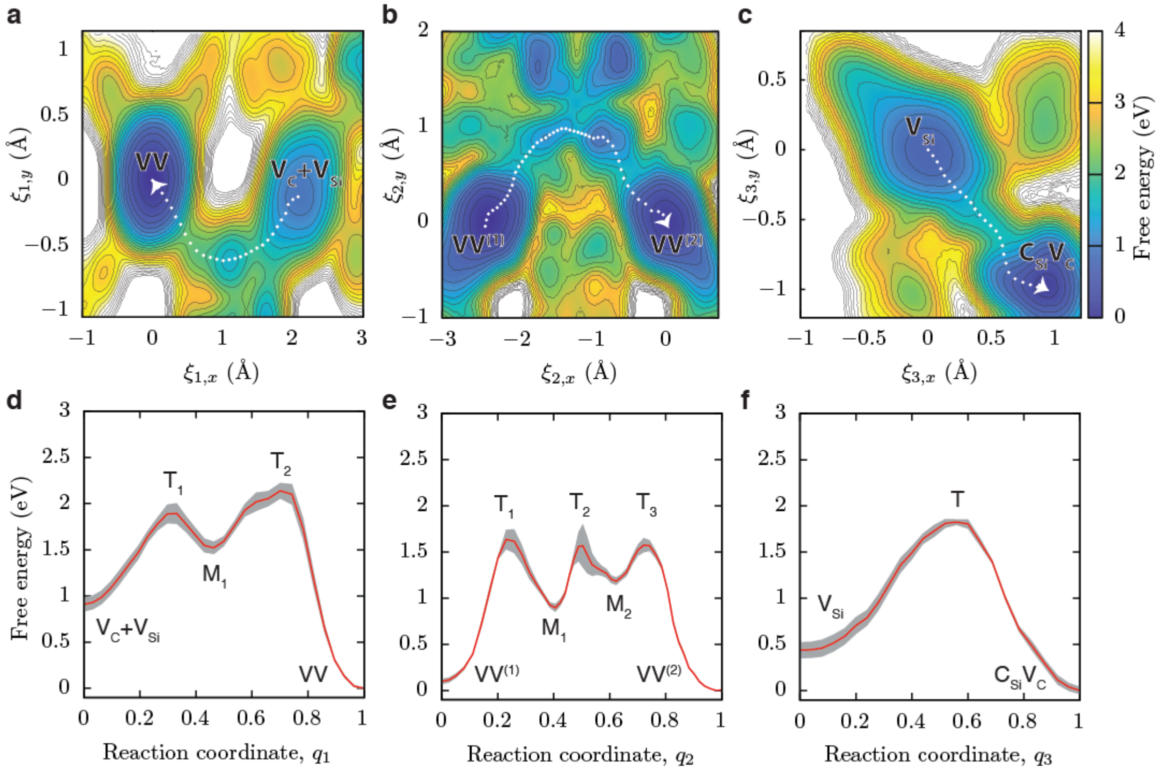

Supplementary Figure 3: Free energy landscapes of vacancy conversion processes for VV and V_{Si} from classical MD. Two-dimensional order parameters, $\xi_i = (\xi_{i,x}, \xi_{i,y})$, are used to describe the position of a carbon atom migrating towards a vacancy site, which is the primary mechanism for VV formation (**a**, **d**), VV reorientation (**b**, **e**), and V_{Si} to $C_{Si}V_C$ conversion processes (**c**, **f**). **a-c**, two dimensional potentials of mean force (PMF) show free energy surfaces of vacancy conversion processes at 1500 K, whose minimum free energy pathways are marked by white dotted lines. **d-f**, Free energy profiles reveal intermediate (state M_i) and transition states (state T_i) along the reaction coordinates. The gray shaded regions denote the error in the PMF determined by block averaging. All three mechanisms are thermally activated processes with relatively high energy barriers (> 1.5 eV).

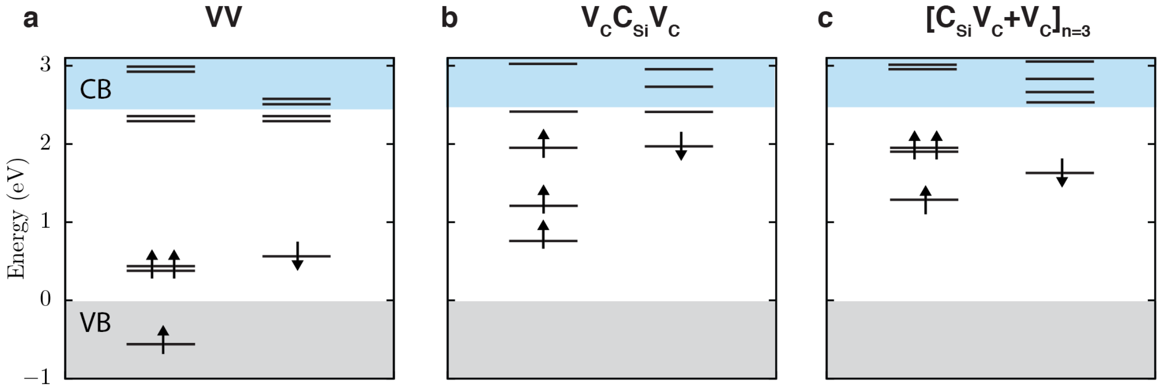

Supplementary Figure 4: Electronic structures of VV^0 and antisite-vacancy complexes obtained using a 216-atom supercell and hybrid-DFT calculations. Defect structures (top) and defect energy level diagrams (bottom) are shown for neutrally charged **a**, VV, **b**, $V_C C_{Si} V_C$, and **c**, $[C_{Si}V_C + V_C]_{n=3}$. Same procedure as in 512-atom supercells (results plotted in Figure 6) was used to compute the electronic structure (see Methods in the main text). The band energy levels computed with smaller supercells also predict a spin triplet ground state.

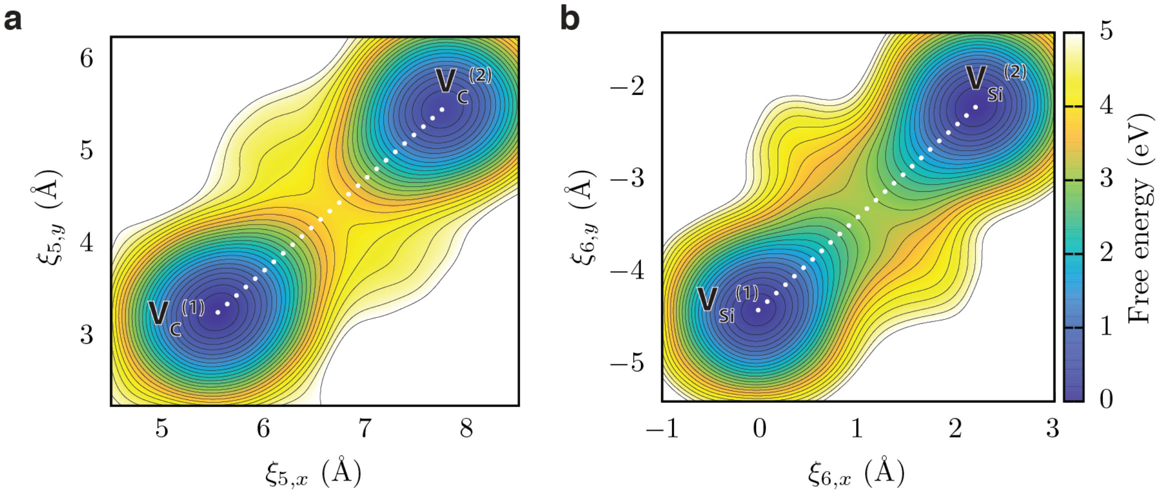

Supplementary Figure 5: Free energy surface for monovacancy migration from FPMD. **a**, 2D PMF of V_C migration via diffusion of a nearest carbon atom, whose cartesian coordinates are used as the collective variables. **b**, 2D PMF of V_{Si} migration via diffusion of a nearest silicon atom, whose cartesian coordinates are used as the collective variables. The MFEPs are shown as white dotted lines. Free energy barriers for migration are ~3.9 eV and ~3.0 eV for V_C and V_{Si}, respectively.

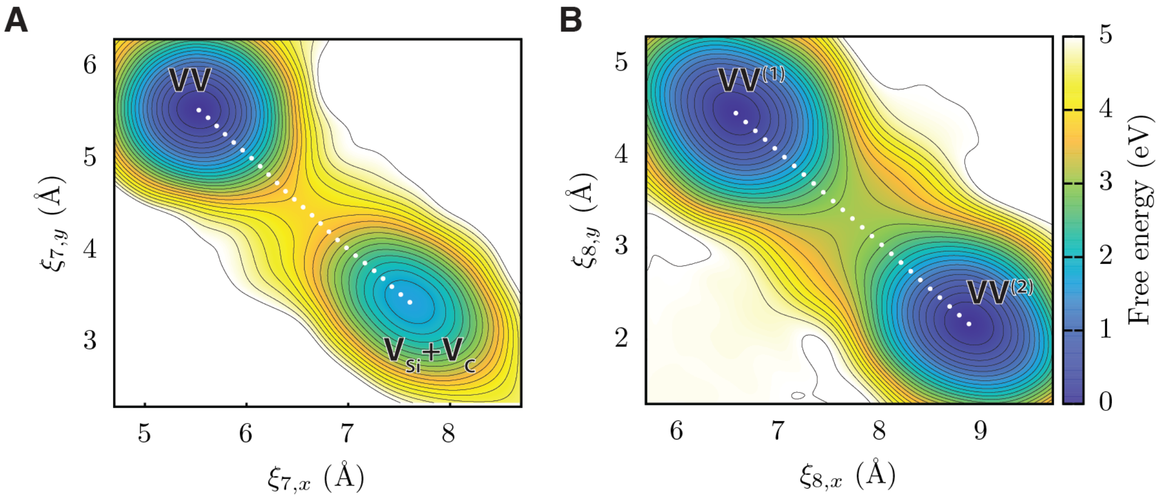

Supplementary Figure 6: Free energy surface for VV formation and VV reorientation from FPMD. a, 2D PMF of VV formation by V_C migration via diffusion of a nearest carbon atom, whose cartesian coordinates are used as the collective variables. **b**, 2D PMF of VV reorientation by V_{Si} migration via diffusion of a nearest silicon atom, whose cartesian coordinates are used as the collective variables. The MFEPs are shown as white dotted lines. Free energy barriers for VV formation and VV reorientation are ~3.9 eV and ~3.1 eV, respectively, which are higher than the other pathways shown in Figs. 3a,3b in the main text.

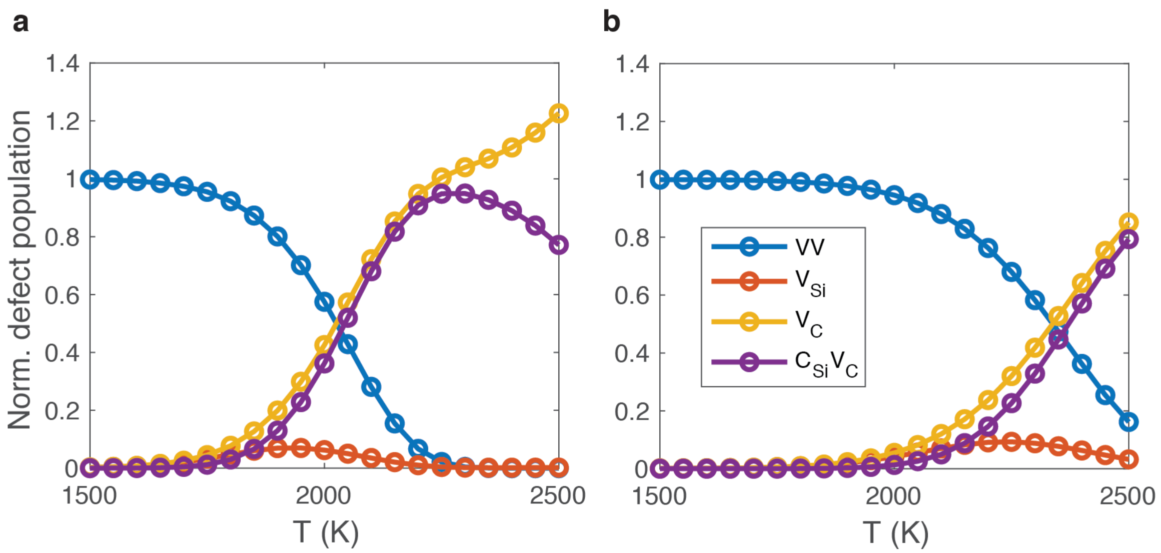

Supplementary Figure 7: Effect of concentration on the kinetics of VV dissociation. Kinetic Monte Carlo simulations for systems with total vacancy concentration of **a**, 1e-3 % and **b**, 1e-4 %.

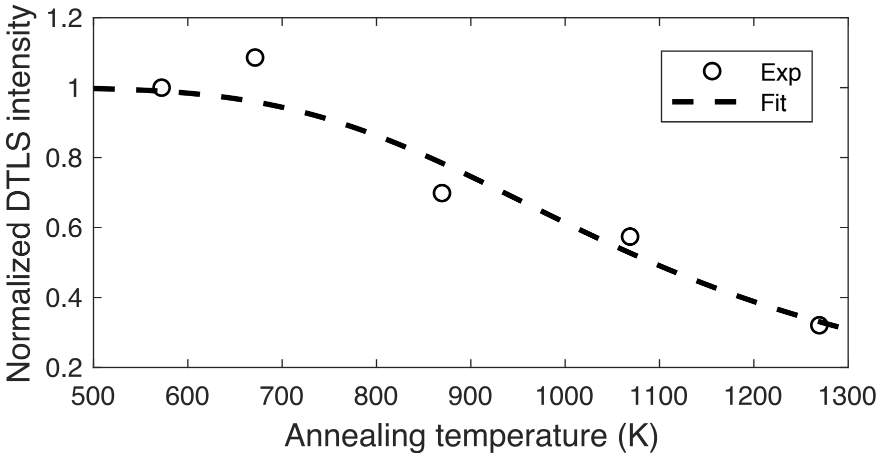

Supplementary Figure 8: Decay in $C_{Si}V_C$ with increasing annealing temperature. Open circles indicate deep level transient spectroscopy (DTLS) measurements of $C_{Si}V_C$ species versus annealing temperature by R. Karsthof, *et al*.[1] Relative population of bound and unbound $C_{Si}V_C$ at T = 1500 K is estimated from the fit to the expression, $y = \frac{1}{1+A\exp(-B/T)}$. The free energy difference is given by $-k_B T \ln P$(bound $C_{Si}V_C$)/P(unbound $C_{Si}V_C$) ~0.2 eV.

Supplementary References

1. Karsthof, R., Bathen, M. E., Galeckas, A. & Vines, L. Conversion pathways of primary defects by annealing in proton-irradiated n-type 4H-SiC. *Phys. Rev. B* **102**, 184111 (2020).
2. Kern, E. L., Hamill, D. W., Deem, H. W. & Sheets, H. D. Thermal properties of β-silicon carbide from 20 to 2000°C. in *Silicon Carbide–1968* S25–S32 (Elsevier, 1969). doi:10.1016/B978-0-08-006768-1.50007-3.
3. Tersoff, J. Modeling solid-state chemistry: Interatomic potentials for multicomponent systems. *Phys. Rev. B* **39**, 5566–5568 (1989).
4. Tersoff, J. Erratum: Modeling solid-state chemistry: Interatomic potentials for multicomponent systems. *Phys. Rev. B* **41**, 3248–3248 (1990).
5. Devanathan, R., Diaz de la Rubia, T. & Weber, W. J. Displacement threshold energies in β-SiC. *J. Nucl. Mater* **253**, 47–52 (1998).
6. Erhart, P. & Albe, K. Analytical potential for atomistic simulations of silicon, carbon, and silicon carbide. *Phys. Rev. B* **71**, 035211 (2005).
7. Gao, F., Bylaska, E. J., Weber, W. J. & Corrales, L. R. Ab initio and empirical-potential studies of defect properties in 3C−SiC. *Phys. Rev. B* **64**, 245208 (2001).
8. Samolyuk, G. D., Osetsky, Y. N. & Stoller, R. E. Molecular dynamics modeling of atomic displacement cascades in 3C–SiC: Comparison of interatomic potentials. *J. Nucl. Mater* **465**, 83–88 (2015).
9. Huang, H., Ghoniem, N. M., Wong, J. K. & Baskes, M. Molecular dynamics determination of defect energetics in beta -SiC using three representative empirical potentials. *Modelling Simul. Mater. Sci. Eng.* **3**, 615–627 (1995).
10. Lucas, G., Bertolus, M. & Pizzagalli, L. An environment-dependent interatomic potential for silicon carbide: calculation of bulk properties, high-pressure phases, point

and extended defects, and amorphous structures. *J. Phys.: Condens. Matter* **22**, 035802 (2010).

11. Pierson, H. O. Characteristics and Properties of Silicon Carbide and Boron Carbide. in *Handbook of Refractory Carbides and Nitrides* 137–155 (Elsevier, 1996). doi:10.1016/B978-081551392-6.50009-X.
