# Supplementary material for: Stability and molecular pathways to the formation of spin defects in silicon carbide
Source: Nat Commun. 2021 Nov 3;12:6325. doi: 10.1038/s41467-021-26419-0 (PMC8566517; doi:10.1038/s41467-021-26419-0)
Supplement: Supplementary file 3 — Description of Additional Supplementary Files [file 41467_2021_26419_MOESM3_ESM.docx]

**File Name:** Supplementary Movie 1

**Description:** MD simulation trajectory showing the V_C_ migration process via a carbon atom diffusing to the V_C_ site at 1500 K

**File Name:** Supplementary Movie 2

**Description:** MD simulation trajectory showing V_Si_ → C_Si_V_C_ by a nearest-neighbor carbon atom hopping to the V_Si_-site, followed by reorientation of C_Si_V_C_ at 1500 K

**File Name:** Supplementary Movie 3

**Description:** MD simulation trajectory showing V_Si_ + V_C_ → VV via a carbon atom diffusing to the V_C_ site at 1000 K.

**File Name:** Supplementary Movie 4

**Description:** MD simulation trajectory showing (1) VV → V_Si_ + V_C_, (2) V_Si_ + V_C_ → VV, and (3) the reorientation of VV at 1500 K.

**File Name:** Supplementary Movie 5

**Description:** MD simulation trajectory showing (1) VV → V_C_C_Si_V_C_, (2) V_C_C_Si_V_C_ → C_Si_V_C_ + V_C_, and (3) the reorientation of C_Si_V_C_ at 1800 K
