# Supplementary figures and images for: Stability and molecular pathways to the formation of spin defects in silicon carbide
Source: Nat Commun. 2021 Nov 3;12:6325. doi: 10.1038/s41467-021-26419-0 (PMC8566517; doi:10.1038/s41467-021-26419-0)

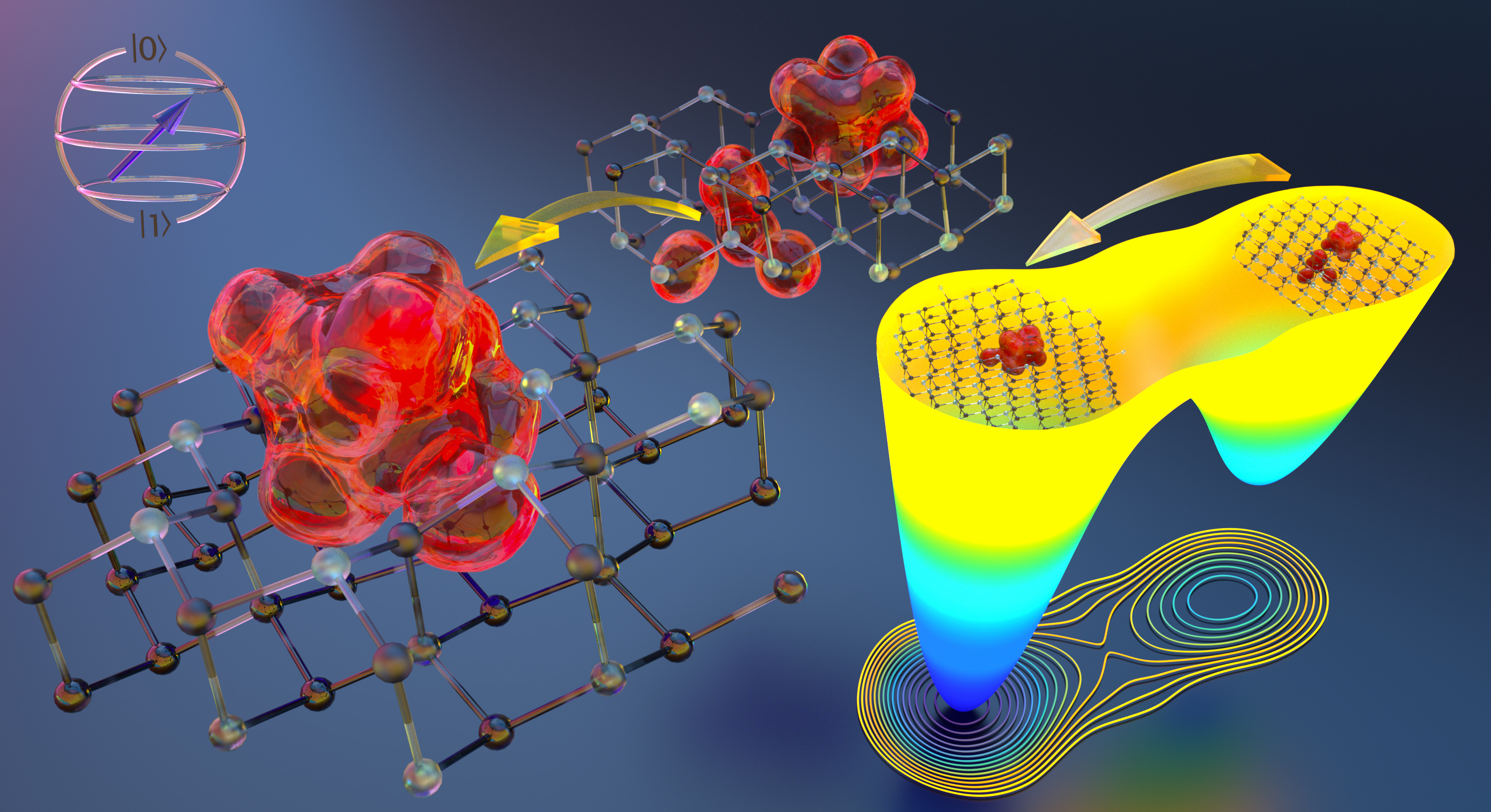

Supplement: Supplementary file 4 — Movie S1 [file 41467_2021_26419_MOESM4_ESM.jpg]

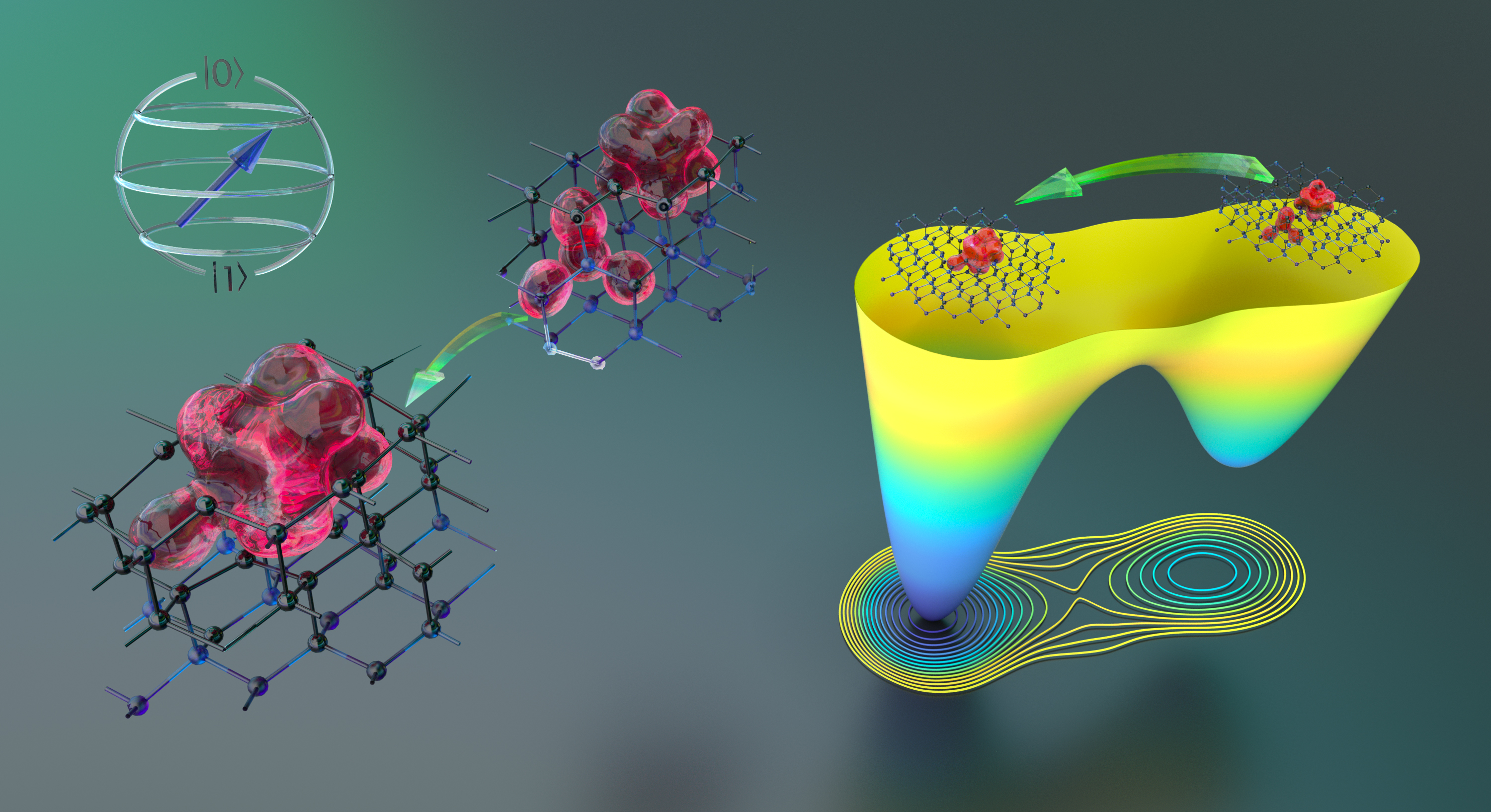

Supplement: Supplementary file 5 — Movie S2 [file 41467_2021_26419_MOESM5_ESM.jpg]

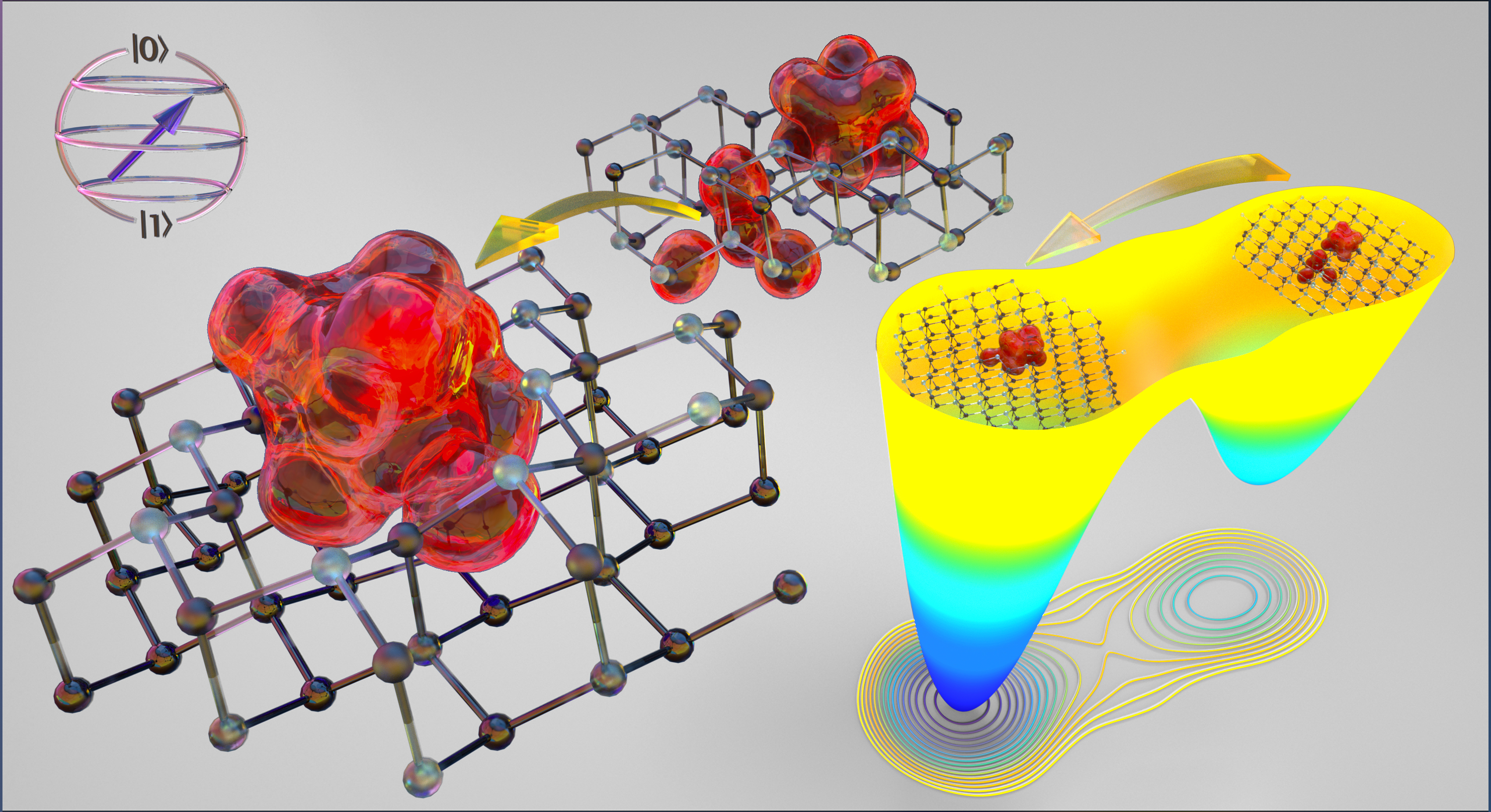

Supplement: Supplementary file 6 — Movie S3 [file 41467_2021_26419_MOESM6_ESM.jpg]

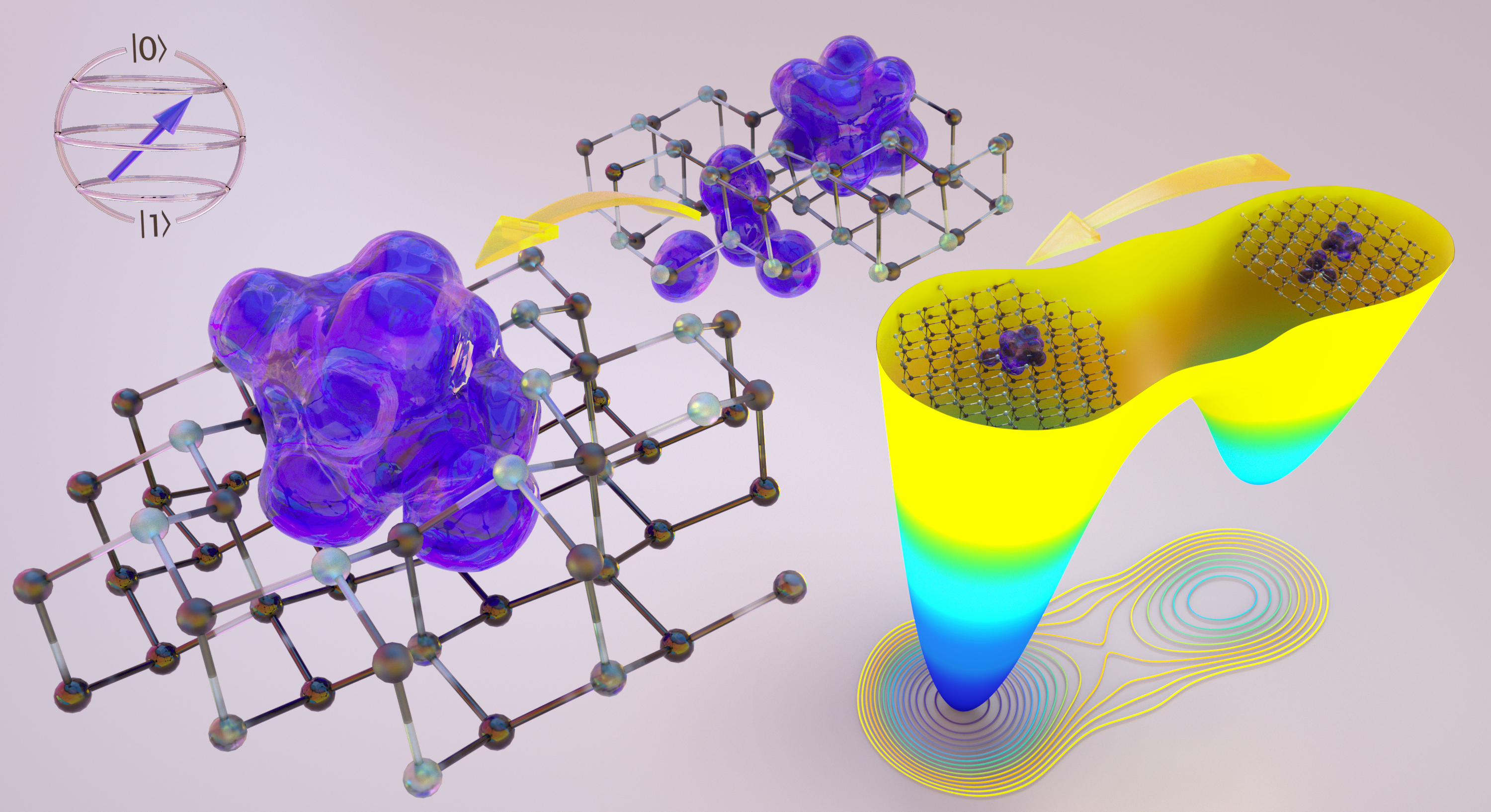

Supplement: Supplementary file 7 — Movie S4 [file 41467_2021_26419_MOESM7_ESM.jpg]
